# A comparative multidimensional LC-MS proteomic analysis reveals mechanisms for furan aldehyde detoxification in *Thermoanaerobacter pseudethanolicus* 39E

**DOI:** 10.1186/s13068-014-0165-z

**Published:** 2014-12-03

**Authors:** Sonya M Clarkson, Scott D Hamilton-Brehm, Richard J Giannone, Nancy L Engle, Timothy J Tschaplinski, Robert L Hettich, James G Elkins

**Affiliations:** BioEnergy Science Center, Oak Ridge National Laboratory, Oak Ridge, TN 37831-6341 USA; Biosciences Division, Oak Ridge National Laboratory, Oak Ridge, TN 37831-6341 USA; Chemical Sciences Division, Oak Ridge National Laboratory, Oak Ridge, TN 37831-6341 USA; Current address: Division of Earth and Ecosystem Sciences, Desert Research Institute, Las Vegas, NV USA

**Keywords:** Thermophiles, Lignocellulosic, Biofuels, Proteomics, Inhibitor, Pretreatment, Furfural, 5-hydroxymethylfurfural, Butanol dehydrogenase

## Abstract

**Background:**

Chemical and physical pretreatment of lignocellulosic biomass improves substrate reactivity for increased microbial biofuel production, but also restricts growth via the release of furan aldehydes, such as furfural and 5-hydroxymethylfurfural (5-HMF). The physiological effects of these inhibitors on thermophilic, fermentative bacteria are important to understand; especially as cellulolytic strains are being developed for consolidated bioprocessing (CBP) of lignocellulosic feedstocks. Identifying mechanisms for detoxification of aldehydes in naturally resistant strains, such as *Thermoanaerobacter* spp., may also enable improvements in candidate CBP microorganisms.

**Results:**

*Thermoanaerobacter pseudethanolicus* 39E, an anaerobic, saccharolytic thermophile, was found to grow readily in the presence of 30 mM furfural and 20 mM 5-HMF and reduce these aldehydes to their respective alcohols *in situ*. The proteomes of *T. pseudethanolicus* 39E grown in the presence or absence of 15 mM furfural were compared to identify upregulated enzymes potentially responsible for the observed reduction. A total of 225 proteins were differentially regulated in response to the 15 mM furfural treatment with 152 upregulated versus 73 downregulated. Only 87 proteins exhibited a twofold or greater change in abundance in either direction. Of these, 54 were upregulated in the presence of furfural and 33 were downregulated. Two oxidoreductases were upregulated at least twofold by furfural and were targeted for further investigation. Teth39_1597 encodes a predicted butanol dehydrogenase (BdhA) and Teth39_1598, a predicted aldo/keto reductase (AKR). Both genes were cloned from *T. pseudethanolicus* 39E, with the respective enzymes overexpressed in *E. coli* and specific activities determined against a variety of aldehydes. Overexpressed BdhA showed significant activity with all aldehydes tested, including furfural and 5-HMF, using NADPH as the cofactor. Cell extracts with AKR also showed activity with NADPH, but only with four-carbon butyraldehyde and isobutyraldehyde.

**Conclusions:**

*T. pseudethanolicus* 39E displays intrinsic tolerance to the common pretreatment inhibitors furfural and 5-HMF. Multidimensional proteomic analysis was used as an effective tool to identify putative mechanisms for detoxification of furfural and 5-HMF. *T. pseudethanolicus* was found to upregulate an NADPH-dependent alcohol dehydrogenase 6.8-fold in response to furfural. *In vitro* enzyme assays confirmed the reduction of furfural and 5-HMF to their respective alcohols.

**Electronic supplementary material:**

The online version of this article (doi:10.1186/s13068-014-0165-z) contains supplementary material, which is available to authorized users.

## Background

Thermophilic bacteria, such as *Clostridium thermocellum* and *Caldicellulosiruptor* species have gained interest for their possible use as biocatalysts for converting lignocellulosic biomass into renewable fuels and chemicals [1-4]. The potential advantages of thermal bioprocessing include improved kinetics, reduced viscosities of concentrated slurries, lower oxygen solubility, and reduced process cooling requirements [2]. In addition, several bacterial phyla include thermophiles that are able to utilize plant cell walls directly through the action of complex (hemi)cellulase systems expressed either as free enzymes, cellulosomes, or multifunctional enzymes [5]. Relying on these native enzymes in a bioprocessing scheme could substantially reduce or even eliminate the need for exogenous enzymes for cellulose solubilization with a resulting improvement in process economics [3,6,7].

To render plant material more reactive to microbial or enzymatic digestion, physical and chemical pretreatment methods are generally applied, and improvement in pretreatment technologies remains a highly active field of research [8,9]. Pretreatment with dilute acid at high temperatures has the benefit of solubilizing the hemicellulose fraction of biomass, which produces fermentable C5 oligomers and monomers [9]. However, one disadvantage of dilute acid pretreatment is that the process is non-specific and, depending on its severity, generates a number of toxic by-products [10]. Inhibitory compounds generated by dilute acid pretreatment typically fall into four categories: organic acids (acetic acid, ferulic acid), ketones (acetovanillone), phenolics (coniferyl alcohol, catechol), and aldehydes (furfural, hydroxymethylfurfural, vanillin). Mixtures of inhibitors, especially those including the furan aldehyde furfural, often have a synergistic effect on inhibiting cell growth and fermentation. For example, furfural increases the toxicity of acetate in yeast [11] and phenols in *Escherichia coli* [12,13]. Furfural is estimated to be responsible for 33% of the toxic effect of sugar cane hydrolysate on *E. coli* LYO1 [14].

In order to compete with more robust ethanologens such as *Saccharomyces cerevisiae*, several limitations inherent to fermentative thermophilic bacteria must be overcome. These limitations include relatively low ethanol titer and yield from mixed-acid fermentation pathways, although breakthroughs in metabolic engineering have improved the yield of ethanol from carbohydrates in some thermophiles to near theoretical limits [15]. Another major hurdle for thermophilic bioprocessing is growth inhibition by a wide range of compounds encountered in biomass fermentations. Insights into overcoming end-product inhibition in *C. thermocellum* have recently emerged [16,17]; however, growth inhibition from other biomass-derived compounds remains underexplored in thermophilic microbes relative to *S. cerevisiae* [18-20] or engineered strains of *E. coli* [12,21-23]. Interestingly, members of the genus *Thermoanaerobacter* have been shown to tolerate pretreated biomass hydrolysates [24,25], and engineered strains give improved ethanol yields from both C5 and C6 sugars [26]. These properties have encouraged the development of several *Thermoanaerobacter* species for bioethanol production from hydrolysates (primarily xylose) and on cellulose when paired with a cellulolytic partner [27]. While surveying thermophilic bacteria for intrinsic tolerance to furfural (unpublished), we observed robust growth and rapid reduction of the compound by *Thermoanaerobacter pseudethanolicus* 39E (formally known as *T. ethanolicus* [28]). This study aims to identify and characterize traits that enable this organism to grow in the presence of and to simultaneously detoxify furan aldehydes through reduction to less toxic alcohols.

## Results and Discussion

### *T. pseudethanolicus* 39E furan aldehyde tolerance

**Figure 1:**
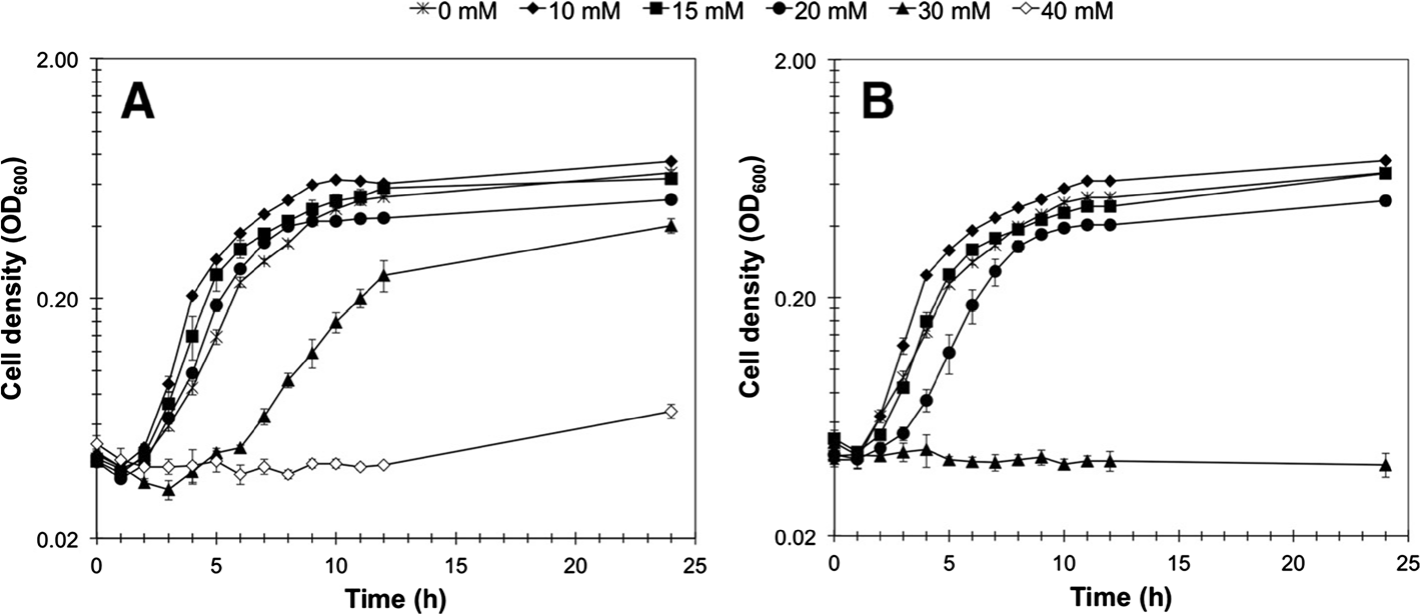
***T. pseudethanolicus*** **furan aldehyde tolerance.** *T. pseudethanolicus* was grown at 65°C with 40 mM glucose and increasing concentrations of **(A)** furfural or **(B)** 5-HMF. Growth was measured by optical density at 600 nm. Error bars are the standard deviation of three replicate cultures.

We initially investigated the growth tolerance of *T. pseudethanolicus* 39E to the furan aldehydes furfural and 5-hydroxymethylfurfural (5-HMF). The addition of 10 mM and 15 mM furfural increased specific growth rates to 0.52 ± 0.03 h^-1^ and 0.49 ± 0.01 h^-1^, respectively, versus the control at 0.38 ± 0.01 h^-1^ (Figure 1A). 5-HMF also stimulated growth at 10 mM compared to no addition (0.51 ± 0.03 versus 0.41 ± 0.02 h^-1^), while growth rates were similar to the control at 15 mM 5-HMF (0.45 ± 0.02 versus 0.41 ± 0.02 h^-1^; Figure 1B). Both 10 mM furfural and 5-HMF slightly increased cell yield at 12 h by approximately 11% and 12%, respectively (Figure 1). Higher growth rates and increased cell yield from the addition of subinhibitory concentrations of furfural and 5-HMF suggest that 39E metabolism is constrained by electron flow, which is relieved by the furan aldehydes serving as an alternative dissimilatory electron acceptor. The concentration resulting in 50% inhibition of growth (IC_50_) with furfural was 30 mM after 12 h and 30 to 40 mM after 24 h. The IC_50_ for 5-HMF was between 20 and 30 mM after both 12 and 24 h. As shown in Table 1, the determined values are comparable to or slightly higher than those of other thermophilic bacteria, while they are higher than reported values for *E. coli*, *S. cerevisiae,* and *Zymomonas mobilis*. Though direct comparisons are difficult due to differences in the growth conditions used in the various studies, these results suggest that *T. pseudethanolicus* 39E has a comparable if not higher tolerance to the furan aldehydes furfural and 5-HMF than other studied organisms*.*

**Table 1:** **IC**_**50**_ (g/L)* for furfural and 5-hydroxymethylfurfural in thermophilic bacteria and ethanologenic microorganisms

**Organism**	**Furfural**	**5-HMF**	**YE (% w/v)**	**Carbon (% w/v)**	**Time (h)**	**Temp (°C)**	**pH**	**Ref.**
***T. pseudethanolicus*** **39E**	**3**	**2 - 3**	**0.1**	**0.7**	**12**	**65**	7.0	TS
***T. pseudethanolicus*** **39E**	**3 - 4**	**2 - 3**	**0.1**	**0.7**	**24**	**65**	7.0	TS
*Bacillus coagulans* MXL-9	2.5 - 5	5	0.5^#^	5-10	24	50	7.0	[29]
*C. saccharolyticus*	1 - 2	1 - 2	0.1	1	16, 40	72	7.0	[30]
*Thermoanaerobacterium* strain AK17^†^	2	3	0.2	0.4	120	60	6.0	[11]
*Tm. thermosaccharolyticum*	1.25	1	0.2	1	40	60	7.0	[31]
*Thermotoga neapolitana*	2 - 4	2 - 4	0.1	1	16, 40	80	7.0	[30]
*E. coli* LY180	1 - 1.5	nd	none	5	48	37	6.5	[23]
*E. coli* LY180	< 0.4	nd	none	5 (xyl)	48	37	6.5	[23]
*E. coli* LY180	1.5	nd	0.1	5 (xyl)	48	37	6.5	[23]
*S. cerevisiae* CBS 1200	0.5	< 1	0.3	2	24	26	5.8	[32]
*S. cerevisiae* NSI 113	2	nd	0.3	1	48	30	5.3	[33]
*Z. mobilis* ATCC 10988	2	5	0.3	2	24	30	5.6	[32]

*Concentration at which 50% inhibition of growth occurred with furfural and 5-hydroxymethylfurfural (5-HMF). Values determined in this study are highlighted in boldface. ^#^Medium also included 1% tryptone; ^†^measured as 50% inhibition of ethanol production; YE, yeast extract; nd, not determined; xyl, xylose; TS, This study.

### Furan aldehyde reduction and glucose fermentation

**Figure 2:**
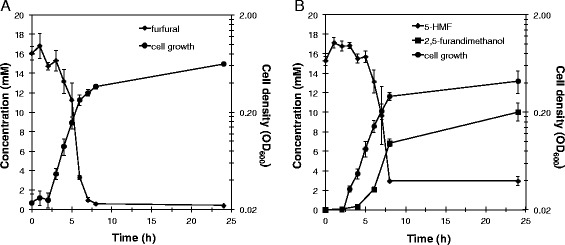
***T. pseudethanolicus*** **furan aldehyde reduction.** *T. pseudethanolicus* was grown at 65°C with 40 mM glucose and 15 mM **(A)** furfural or **(B)** 5-HMF. Growth was measured by optical density at 600 nm. Furan aldehyde concentration was measured spectrophotometrically, while furan alcohol concentration was measured by gas chromatography-mass spectrometry. Error bars are the standard deviation of three replicate cultures.

In order to establish the mechanism of increased furan aldehyde tolerance, *T. pseudethanolicus* 39E was grown in the presence and absence of 15 mM furfural or 5-HMF and the furan aldehyde and respective furan alcohol concentrations were measured. As shown in Figure 2, concomitant with growth, furfural and 5-HMF concentrations decreased while 2,5-furandimethanol concentration increased, indicating that *T. pseudethanolicus* 39E reduced 5-HMF to 2,5-furandimethanol. Furfural was most likely also reduced to furfuryl alcohol; however, quantitation of this compound is complicated by its polymerization at the growth temperature of *T. pseudethanolicus* 39E*.*

**Figure 3:**
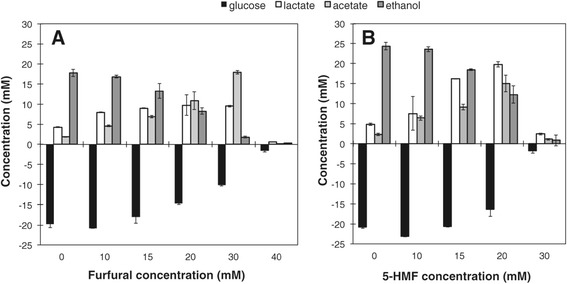
**24-h substrate and end product analysis.** *T. pseudethanolicus* was grown at 65°C with 40 mM glucose and increasing concentrations of **(A)** furfural or **(B)** 5-HMF for 24 h. Culture supernatants were analyzed for glucose and end product formation by HPLC. Glucose consumption was determined by comparison with an uninoculated control. Error bars are the standard deviation of three replicate cultures.

The addition of furan aldehydes affected end-product formation by *T. pseudethanolicus* 39E, as determined by HPLC analysis (Figure 3). At 10 mM concentrations, more acetate and lactate are produced, while ethanol production remains constant. At furan aldehyde concentrations above 10 mM where growth is observed (15, 20, 30 mM furfural and 15, 20 mM 5-HMF), ethanol decrease and acetate increase are directly proportional, suggesting that acetyl coenzyme A is converted to acetate through phosphotransacetylase (PTA) and acetate kinase (AK) rather than serving as an electron acceptor for ethanol production via NAD(P)H-dependent bifunctional alcohol dehydrogenase (ADH) activity. *T. pseudethanolicus* 39E possesses seven ADHs, but ethanol is primarily produced from NADPH-dependent AdhB [34-36]. The oxidative branch of the pentose phosphate pathway is also present in 39E, which could supply NADPH [34]. Reduction of furfural/5-HMF to their corresponding alcohols during growth competes with ethanol production for electrons delivered by NADPH. This is also suggested by stoichiometric shifts in end products, where added aldehydes resulted in about a 0.5 times decrease in corresponding molar ethanol concentrations (that is, the 30 mM furfural addition resulted in a decrease of 15 mM ethanol versus the control). This shift in ethanol/acetate concentrations is consistent with an electron balance of one NAD(P)H per furan aldehyde and two NAD(P)H per ethanol. The reason for increased lactate with added furan aldehyde, especially 5-HMF, is less clear. Further redox imbalances from the presence of furfural or 5-HMF may direct more NADH generated from glycolysis to be oxidized via lactate dehydrogenase (LdhA). Furfural addition to a growing culture of the related thermophilic bacterium *C. thermocellum* also resulted in increased lactate production and cessation of ethanol production [37], although the reason for these changes is unknown. The mesophilic ethanologen *S. cerevisiae* has also been shown to remove furan aldehydes by reduction to their respective alcohols at the expense of ethanol production [38]. This has been shown to involve an upregulation of central carbon metabolism, especially the NADPH-generating pentose phosphate pathway [39], and downregulation of enzymes involved in ethanol formation, thereby increasing the availability of reducing equivalents for aldehyde detoxification [40].

### Cell-wide proteomic response to furfural

**Figure 4:**
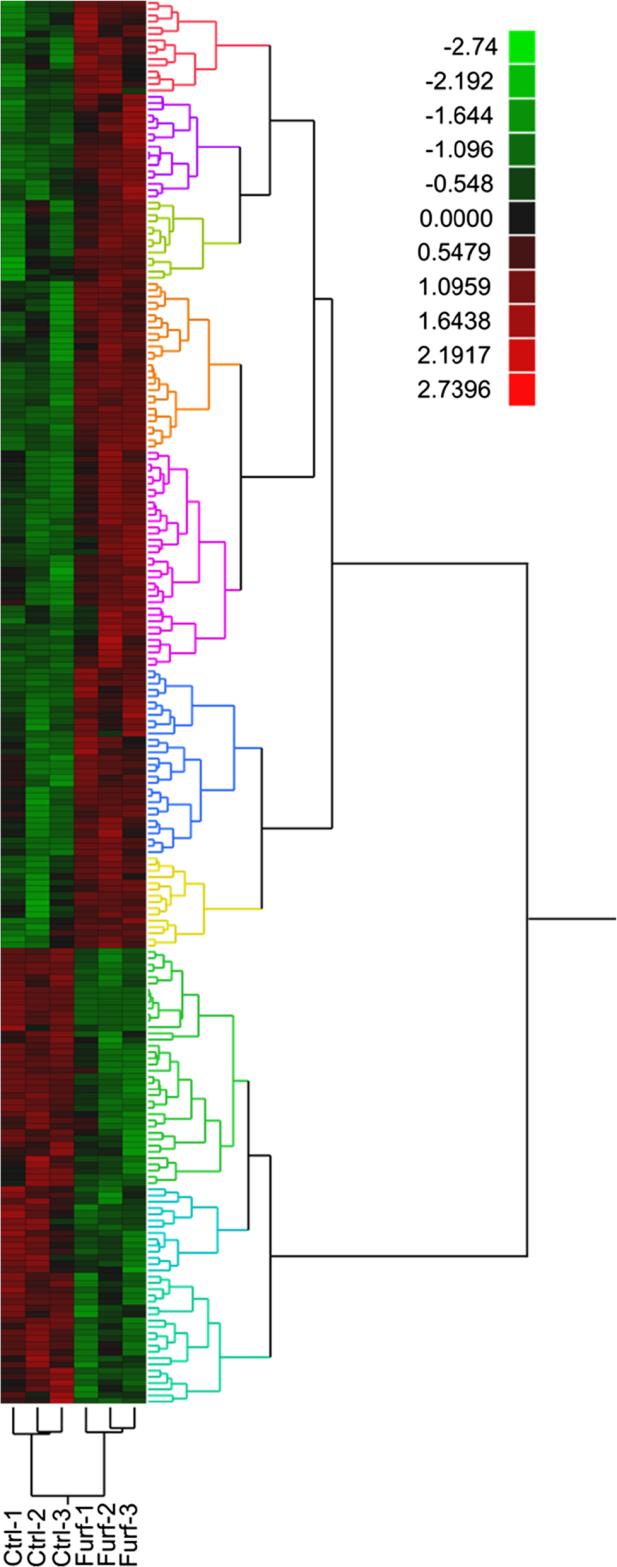
**Heat map of proteomic analysis with and without 15 mM furfural.** Proteins exhibiting a statistically significant (ANOVA, *P* ≤0.05) difference in abundance are included. Each protein (row) was independently normalized to recast spectral count values as standard deviations from the row mean. Protein abundance differences were clustered according to trends measured across all biological replicates. Red = increased; green = decreased abundance.

In order to identify potential enzymes involved in reducing furfural and 5-HMF and also to examine cell-wide physiological effects of furan aldehyde exposure, proteomic analysis was performed comparing *T. pseudethanolicus* 39E grown with and without furfural. A concentration of 15 mM furfural was used to challenge the cells since this was the maximum amount that displayed a stimulatory effect during the growth experiments (Figure 1A). Triplicate cultures were grown in parallel either in the presence or absence (control) of furfural and harvested at 8 h after inoculation, which corresponded to the time interval necessary for complete reduction of furfural to furfuryl alcohol (Figure 2A). Peptide samples were prepared and analyzed as described in the Methods section. The mass spectrometry proteomics data have been deposited to the ProteomeXchange Consortium [41] via the PRIDE partner repository with the dataset identifier PXD001446. The complete proteomics dataset and statistical analysis are also provided in Additional file 1: Table S1, which includes an in-table, color-coded heat map corresponding to Figure 4. In total, 1,294 proteins were identified across both conditions with roughly 300,000 spectra (SpC) assigned to constituent peptides. Of the 1,294 proteins, only 918 passed the 99% SpC cutoff and were moved to the ANOVA analysis. Using a *P*-value cutoff of 0.05, 225 proteins were found to be differentially expressed with 152 upregulated in furfural treated cells versus 73 downregulated. Culling this list even further, only 87 proteins exhibited a twofold or greater change in abundance in either direction. Of these, 54 were upregulated in the presence of furfural (Table 2) and 33 were downregulated (Table 3). Significantly regulated proteins were grouped into 11 clusters based on abundance pattern across all replicates (Figure 4). Many cellular functions were affected by furfural, with the most highly downregulated proteins involved in cell wall biosynthesis or sporulation. Hydrogenase-related proteins were also downregulated, along with several redox proteins predicted to use NAD(P)H. Upregulated proteins fell into 12 general cellular functions, with those regulated fivefold or higher falling into three categories: polar amino acid biosynthesis (arginine, cysteine), nucleotide metabolism, and redox proteins.

**Table 2:** **Proteins upregulated twofold or more by 15 mM furfural (** ***P*** **≤0.05)**

**Locus**	**Fold change**	**Description**	***P*** **-value**
**Amino acid biosynthesis**			
Teth39_0141	2.22	Threonine synthase	0.015
Teth39_0223	4.31	N-acetyl-gamma-glutamyl-phosphate reductase	0.001
**Teth39_0224**	**6.61**	**Arginine biosynthesis bifunctional protein ArgJ**	**0.000**
Teth39_0225	2.50	Acetylglutamate kinase	0.010
Teth39_0226	2.65	Acetylornithine and succinylornithine aminotransferase	0.038
**Teth39_0227**	**7.69**	**Carbamoyl phosphate synthase, small subunit**	**0.010**
**Teth39_0228**	**12.73**	**Carbamoyl phosphate synthase, large subunit**	**0.008**
**Teth39_0229**	**5.74**	**Argininosuccinate synthase**	**0.000**
Teth39_0279	3.50	Cysteine synthase A	0.001
**Teth39_0280**	**6.68**	**Cysteine desulfurase**	**0.001**
**Teth39_0281**	**9.01**	**tRNA methyltransferase**	**0.014**
Teth39_0559	2.56	Diaminopimelate decarboxylase	0.001
Teth39_0983	3.14	Prephenate dehydratase	0.002
Teth39_1666	2.17	Glutamine synthetase, catalytic region	0.003
Teth39_1810	3.56	Aspartate 1-decarboxylase	0.033
**Carbohydrate metabolism**			
Teth39_0611	2.28	Beta-galactosidase	0.011
Teth39_0744	2.26	Mannose-6-phosphate isomerase, class I	0.034
Teth39_1512	3.34	Kojibiose phosphorylase	0.043
**Cell division/sporulation/motility**			
Teth39_1000	2.05	SpoIID/LytB domain	0.009
Teth39_1257	4.17	Flagellar M-ring protein FliF	0.043
Teth39_1280	2.08	Chromosome segregation protein SMC	0.008
**Chaperones**			
Teth39_0115	2.15	Chaperonin Cpn10	0.013
Teth39_1392	2.21	Chaperone protein DnaJ	0.005
**Energy related**			
Teth39_1820	2.04	Homocitrate synthase	0.003
Teth39_2064	6.75	2-hydroxyacid dehydrogenase, NAD-binding	0.019
**Hypothetical proteins**			
Teth39_0463	2.28	Hypothetical protein	0.012
Teth39_0919	2.78	Hypothetical protein	0.023
**Iron-sulfur cluster metabolism**			
Teth39_0116	2.14	FeS assembly ATPase SufC	0.020
Teth39_0117	2.65	FeS assembly protein SufB	0.001
Teth39_0118	2.97	SufBD protein	0.000
Teth39_0120	2.66	SUF system FeS assembly protein, NifU family	0.001
**Nucleotide related**			
Teth39_0775	2.04	MutS2 family protein	0.005
Teth39_1049	2.07	tRNA methyltransferase	0.018
Teth39_1323	2.46	DNA-directed RNA polymerase, omega subunit	0.005
Teth39_1713	2.12	Phosphoribosylformylglycinamidine synthase II	0.006
**Teth39_1803**	**36.39**	**Phosphoribosylaminoimidazole carboxylase, ATPase subunit**	**0.000**
Teth39_1828	2.76	Cytidine deaminase	0.009
Teth39_1832	2.14	ABC transporter related	0.004
**Redox proteins**			
Teth39_0646	3.61	FAD-dependent pyridine nucleotide disulfide oxidoreductase	0.005
Teth39_0720	3.77	4Fe-4S ferredoxin, iron-sulfur binding domain protein	0.044
**Teth39_1597**	**6.84**	**Iron-containing alcohol dehydrogenase**	**0.000**
**Teth39_1598**	**6.32**	**Aldo/keto reductase**	**0.000**
**Ribosomal proteins**			
Teth39_0365	2.00	Ribosomal protein L7/L12	0.020
Teth39_1753	2.01	RNA binding S1 domain protein	0.001
Teth39_2275	2.33	Ribosomal protein S6	0.006
**Transporters**			
Teth39_0231	2.85	Calcium translocating P-type ATPase, PMCA-type	0.001
Teth39_0278	2.82	Heavy metal translocating P-type ATPase	0.036
Teth39_0282	4.65	Copper translocating P-type ATPase	0.004
Teth39_1033	2.26	Efflux transporter, RND family, MFP subunit	0.015
Teth39_1765	4.62	Extracellular solute-binding protein, family 3	0.001
Teth39_2232	2.43	Type IV secretory pathway VirB4 components-like protein	0.011
**Vitamin related**			
Teth39_0307	3.10	Biotin/lipoyl attachment domain-containing protein	0.007
Teth39_1205	2.03	Riboflavin biosynthesis protein RibF	0.001
Teth39_1559	2.00	SNO glutamine amidotransferase	0.001

Sorted by general cellular function. Proteins in boldface are regulated greater than fivefold. Descriptions are from the National Center for Biotechnology Information.

**Table 3:** **Proteins downregulated twofold or more by 15 mM furfural (** ***P*** **≤0.05)**

**Locus**	**Fold change**	**Description**	***P*** **-value**
**Amino acid metabolism**			
Teth39_0216	-3.10	Glutamate synthase, homotetrameric	0.000
Teth39_0217	-2.47	Oxidoreductase FAD/NAD(P)-binding domain	0.004
Teth39_0487	-2.15	Alanine racemase	0.038
Teth39_1661	-3.77	Glutamine amidotransferase, class II	0.016
Teth39_2007	-2.58	Aromatic amino acid beta-eliminating lyase	0.004
**Cell division/sporulation/motility**			
**Teth39_0175**	**-52.70**	**Peptidoglycan-binding LysM**	**0.000**
**Teth39_0252**	**-30.04**	**YabP family protein**	**0.000**
Teth39_1446	-2.42	Cell division topological specificity factor MinE	0.016
Teth39_1772	-2.33	Flagellar protein FlaG protein	0.011
Teth39_1783	-2.51	Flagellar hook-associated protein 3	0.009
**Energy related**			
Teth39_0466	-2.27	Thiamine pyrophosphate enzyme domain	0.038
**Hydrogenase related**			
Teth39_0221	-2.17	Hydrogenase with PAS/PAC sensor	0.005
Teth39_1458	-2.05	Hypothetical protein	0.005
**Teth39_1459**	**-24.50**	**Histidine kinase**	**0.000**
**Hypothetical proteins**			
**Teth39_0794**	**-5.93**	**Hypothetical protein**	**0.041**
**Teth39_0842**	**-36.68**	**Hypothetical protein**	**0.000**
**Nucleotide related**			
Teth39_1357	-2.51	Metal-dependent phosphohydrolase	0.002
**Teth39_2157**	**-9.29**	**SirA family protein**	**0.000**
**Redox proteins**			
Teth39_0445	-2.19	Thioredoxin reductase	0.002
Teth39_1916	-2.73	Oxidoreductase FAD/NAD(P)-binding domain	0.001
Teth39_1917	-4.88	4Fe-4S ferredoxin, iron-sulfur binding domain	0.000
Teth39_2155	-4.30	FAD-dependent pyridine nucleotide-disulfide oxidoreductase	0.000
**Transcriptional regulator**			
Teth39_0150	-2.56	Transcriptional regulator, DeoR family	0.011
Teth39_0757	-3.07	Putative cold-shock DNA-binding domain protein	0.029
Teth39_1109	-2.27	Sporulation transcriptional activator Spo0A	0.006
Teth39_1292	-2.06	Hypothetical protein	0.019
Teth39_1796	-2.17	Two-component transcriptional regulator, winged helix family	0.015
**Transporters**			
Teth39_0333	-2.98	PTS system, fructose subfamily, IIC subunit	0.001
Teth39_0334	-2.90	PTS system, fructose-specific, IIB subunit	0.019
**Vitamin related**			
Teth39_0787	-4.71	Lipoic acid synthetase	0.033
**Other**			
Teth39_0542	-2.43	Dihydroxyacetone kinase, DhaK subunit	0.006
Teth39_1065	-2.21	HAD superfamily (subfamily IIIA) phosphatase, TIGR01668	0.024
Teth39_1216	-2.04	1-hydroxy-2-methyl-2-(E)-butenyl 4-diphosphate synthase	0.011

Sorted by general cellular function. Proteins in boldface are regulated greater than fivefold. Descriptions are from the National Center for Biotechnology Information.

#### Energy production and carbohydrate metabolism

Comparatively, the proteomic response of *T. pseudethanolicus* 39E to furfural showed similarities at the functional level to the responses of *C. thermocellum*, as well as *S. cerevisiae* and *E. coli*. While central carbon metabolism did not appear to be significantly impacted by furfural in *T. pseudethanolicus* 39E, upregulated carbohydrate-related proteins included beta-galactosidase, mannose-6-phosphate isomerase, and kojibiose phosphorylase, while dihydroxyacetone kinase and two fructose-specific, phosphoenolpyruvate-dependent sugar phosphotransferase system transport proteins were downregulated. Genes involved in energy production and conversion, as well as carbohydrate transport and metabolism, were also regulated in *C. thermocellum* ATCC 27405 [37]. Acetate kinase and phosphoacetyltransferase were both downregulated, though this is likely a general stress response rather than furfural-specific. As in *T. pseudethanolicus* 39E, beta-galactosidase was upregulated in *C. thermocellum* ATCC 27405 upon furfural exposure, as were several glycosyl transferase family proteins, though the reason for this regulation is unclear. On the other hand, central carbon metabolism is significantly upregulated in both the *S. cerevisiae* and *E. coli* response to furfural. In anaerobic *S. cerevisiae* fermentations, an 8 g/L furfural treatment repressed the synthesis of enzymes involved in glucose catabolism and the tricarboxylic acid (TCA) cycle. Conversely, addition of 17 g/L furfural to an aerobic *S. cerevisiae* culture increased expression of proteins involved in glycolysis and the TCA cycle, while repressing expression of proteins involved in glycerol and ethanol production [42]. Analysis of a single-gene disruption library of *S. cerevisiae* BY4741 against growth with furfural identified several genes in the pentose phosphate pathway as important in furfural tolerance [39], especially *ZWF1*, whose overexpression allowed for growth with 50 mM furfural. In an ethanologenic strain of *E. coli* (LY180), a moderate furfural challenge (0.5 g/L) perturbed the expression of about 400 genes at least twofold, 15 min after exposure, with central carbon and energy metabolism being among the pathways regulated [22]. It is interesting to note that central carbon metabolism is significantly regulated in the mesophilic *S. cerevisiae* and *E. coli* and is much less affected in the thermophilic *C. thermocellum* and *T. pseudethanolicus*, though the reason for this difference remains unclear.

#### Stress response

With 15 mM furfural, *T. pseudethanolicus* 39E did not display a typical stress phenotype indicated by a reduced growth rate. Nevertheless, some functions associated with stress were differentially regulated. The expression of eight predicted transporters was affected by furfural, including upregulation of three metal transporters and one efflux transporter. A variety of nucleotide-related genes were also upregulated, including two *de novo* purine biosynthesis genes (Teth39_1713 and Teth39_1803) and two genes involved in nucleoside degradation (Teth39_1828 and Teth39_1832). Additionally, Teth39_1216, predicted to be involved in isoprenoid biosynthesis, and Teth39_0175, predicted to function in cell wall turnover, are downregulated in response to furfural. Similarly, a number of stress responses are upregulated by furfural in *C. thermocellum* ATCC 27405, including many genes that are homologous to class I and class IV heat shock response genes in *Bacillus subtilis*, though these genes were also upregulated by heat [37] and ethanol treatment [43]. A number of uncharacterized transporters were also regulated, as well as genes involved in transcription, RNA processing and modification, chromatin structure and dynamics, and DNA replication, recombination, and repair. In *S. cerevisiae*, stress responses upregulated by furfural include osmotic and salt stress, DNA damage, and pH stress [40]. *S. cerevisiae* also responds to furan aldehydes by regulating cell adaptation and survival processes, especially with respect to drug resistance, transport, and cell membrane composition [38]. In *E. coli* LY180, transport functions, as well as cell structure, DNA, and lipid synthesis functions are also regulated by furfural [22]. A general stress response to furfural thus appears to include upregulation of transport functions and nucleotide metabolism.

#### Amino acid metabolism

In *T. pseudethanolicus* 39E grown with 15 mM furfural, both cysteine and arginine biosynthetic genes were upregulated (cysteine synthase A, Teth39_0279; cysteine desulfurase, Teth39_0280; arginine biosynthesis, Teth39_0223-0229). Amino acid metabolism is also affected by furfural stress in *C. thermocellum*, *S. cerevisiae,* and *E. coli*. In *C. thermocellum* ATCC 27405*,* arginine biosynthetic genes are upregulated upon furfural addition [37]. While sulfur amino acid biosynthesis is not directly regulated, genes involved in sulfate transport and sulfur assimilation are upregulated by furfural. In *S. cerevisiae*, proteins involved in sulfur amino acid biosynthesis are downregulated upon exposure to 8 g/L furfural under anaerobic conditions [40]. In *E. coli* LY180, 0.5 g/L furfural also repressed genes involved in arginine biosynthesis, but induced expression of sulfur-containing amino acid biosynthetic genes [22]. This is due to a decrease in NADPH availability, which is instead used by the aldehyde reductases YqhD and DkgA for furfural reduction [22]. Upregulation of cysteine biosynthetic pathways in *T. pseudethanolicus* 39E suggests that, as in *E. coli*, increased expression of NAD(P)H-dependent aldehyde reductases (described below) may decrease NADPH availability for sulfur amino acid biosynthesis.

#### Redox metabolism

Six alcohol dehydrogenases (ADHs) identified in *T. pseudethanolicus* 39E were differentially regulated. The three functionally characterized ADHs [36,44], AdhA (Teth39_0220), AdhB (Teth39_0218), and AdhE (Teth39_0206), were all downregulated (*P* <0.05); however, none more than twofold (AdhA 1.27-fold, AdhB 1.81-fold, and AdhE 1.59-fold). Of the other three identified alcohol dehydrogenases in *T. pseudethanolicus* 39E (Teth39_0878, Teth39_1597, Teth39_1979), only Teth39_1597 was significantly upregulated (6.8-fold; *P* <0.001). Another oxidoreductase upregulated by *T. pseudethanolicus* 39E in response to furfural is Teth39_1598 (6.3-fold; *P* <0.001). These genes potentially encode enzymes involved in reducing furfural and 5-HMF and will be discussed in more detail below. In *C. thermocellum* ATCC 27405*,* the bifunctional alcohol/aldehyde dehydrogenase Cthe_0423 and the redox regulator Rex (Cthe_0422) were downregulated upon furfural addition [37]. No other alcohol dehydrogenase or aldehyde reductase was differentially regulated; however, a putative carbon monoxide dehydrogenase (Cthe_0281) was upregulated by furfural and may play a role in redox balance in *C. thermocellum* ATCC 27405 [37]. In *S. cerevisiae*, NAD(P)H-dependent aldehyde reductases or alcohol dehydrogenases (ADH) have also been shown to affect furan aldehyde tolerance [45] and be regulated by furan aldehydes [40]. Transcriptomic expression analysis of known reductase and dehydrogenase genes showed that *ADH2* was highly expressed in hydrolysate-tolerant *S. cerevisiae* strain TMB3000 compared to the wild-type CBS8066 and was also induced by 5-HMF [45]. In a proteomic analysis of the response of *S. cerevisiae* to 17 g/L furfural, six ADHs showed differential regulation, with Adh1p, Adh5p, and Adh6p upregulated, Adh2p and Sfa1p downregulated, and Adh4p unregulated by furfural [42]. Adh6p and Adh7p have furfural and 5-HMF reductase activity, with the former using both NADH and NADPH and the latter only NADH [46]. In *E. coli* LY180, energy functional groups are also highly regulated [22]. As in *S. cerevisiae,* NADPH-dependent aldehyde reductases with furfural reducing capacity are upregulated, namely *yqhD* and *dkgA*.

### Enzyme cloning and activity measurements

Teth39_1597 [GenBank GeneID:5874751] shares 36% identity/54% similarity with *yqhD* from *E. coli* LY180 [22]. Teth39_1597 belongs to the Fe-dependent alcohol dehydrogenase superfamily (pfam00465) with predicted butanol dehydrogenase activity (BDH, cd08187). The gene product appears to be a close homolog of BdhA in *Thermoanaerobacter mathranii* (88% identity/94% similarity), which has been experimentally verified to have BDH activity [47]. Thus, Teth39_1597 is considered to be a butanol dehydrogenase and will be referred to as Teth39 *bdhA*. Teth39_1598 [GenBank GeneID:5874752] has 27% identity/41% similarity to *dkgA* from *E. coli* LY180, another enzyme shown to have NADPH-dependent furfural reductase activity [22]. Teth39_1598 is a predicted aldo/keto oxidoreductase and will be referred to as Teth39 *akr*.

**Figure 5:**
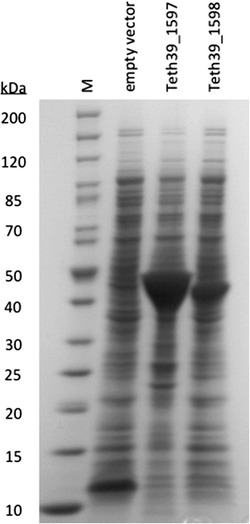
**Overexpression of Teth39_1597 and Teth39_1598 in** ***Escherichia coli*** **.** Teth39_1597 (48.4 kDa) and Teth39_1598 (42.1 kDa) were cloned from *T. pseudethanolicus* into pET-30a and overexpressed from a T7 promoter after induction with IPTG. Overexpression was determined in whole cell extract by Coomassie-stained SDS-PAGE.

Since both Teth39 *bdhA* and Teth39 *akr* were significantly upregulated in response to furfural and are homologs to similarly upregulated *E. coli* genes *yqhD* and *dkgA*, further biochemical characterization was performed to determine their cofactor and substrate specificities. The coding regions for Teth39 *bdhA* and Teth39 *akr* were PCR amplified from *T. pseudethanolicus* 39E genomic DNA and cloned into pET-30a behind a T7-*lac* promoter and N-terminal 6xHis- and S-tags. Overexpression plasmids, as well as the pET-30a plasmid alone, were transformed into *E. coli* BL21 (DE3) and Teth39 BdhA and Teth39 AKR were overexpressed (Figure 5). Whole cell lysates were prepared and assayed for aldehyde reductase activity aerobically at 60°C with acetaldehyde, furfural, and 5-HMF. Furfural was also assayed under anaerobic conditions. Teth39 BdhA and Teth39 AKR activities were compared to the vector-only control (Table 4). Neither enzyme showed any activity with NADH as cofactor. Teth39 AKR had minimal activity with both furan aldehydes using NADPH as the cofactor, but not above the vector control. In contrast, Teth39 BdhA showed activity above the vector control using NADPH as the cofactor with both furan aldehydes. The specific activity was 4.97 ± 0.17 U with furfural and 10.06 ± 0.80 U with 5-HMF.

**Table 4:** **Specific activity (μmol/min/mg protein) of putative** ***T. pseudethanolicus*** **39E aldehyde reductases**

	**Vector control**	**Teth39 BdhA**	**Teth39 AKR**
Furfural	0.33 ± 0.13	**4.97 ± 0.17**	0.27 ± 0.04
5-Hydroxymethylfurfural	0.44 ± 0.16	**10.06 ± 0.80**	0.39 ± 0.07
Acetaldehyde	0.02 ± 0.15	**4.54 ± 0.76**	0.02 ± 0.01
Butyraldehyde	0.09 ± 0.05	**14.58 ± 3.57**	**0.33 ± 0.04**
Isobutyraldehyde	−0.10 ± 0.21	**0.63 ± 0.29**	**0.23 ± 0.05**

Whole cell lysate with pET-30a only (vector control) or expressing Teth39 BdhA (Teth39_1597) or Teth39 AKR (Teth39_1598) was assayed aerobically at 60°C using 0.2 mM NAD(P)H as the electron donor and 20 mM substrate. NAD(P)H oxidation activity was measured via loss of absorbance at 340 nm. Values in boldface are statistically significant. No activity was detected with either Teth39 BdhA or Teth39 AKR above vector control using NADH.

Based on these results, overexpression of Teth39 *bdhA* may increase microbial tolerance to the furan aldehydes furfural and 5-HMF produced during biomass pretreatment, especially in a thermophilic, cellulolytic microbe, such as a *Caldicellulosiruptor* sp. or *Clostridium thermocellum*. This approach has proven successful in *S. cerevisiae,* where overexpression of *ADH6* or *ADH7* allowed growth with 40 mM 5-HMF, where none was seen in a control [46]. However, the increased expression of Teth39 *bdhA* in *T. pseudethanolicus* 39E may have influenced other aspects of its furfural proteomic response. For example, Teth39 BdhA is predicted to contain iron-sulfur clusters, and iron-sulfur cluster biosynthetic genes (Teth39_0116-0120) are also upregulated by furfural. Additionally, other oxidoreductases predicted to use NADPH as a cofactor are downregulated (Teth39_0216, Teth39_0217, Teth39_1916), perhaps to increase NADPH availability for furfural reduction. Thus, while overexpression of Teth39 BdhA alone may increase furan aldehyde tolerance, coexpression of other genes may be required to achieve the phenotype overall.

Teth39 BdhA and Teth39 AKR also exhibited butyraldehyde and isobutyraldehyde reduction activity (Table 4), presumably forming butanol and isobutanol, with the Teth39 BdhA activity 2.7-fold higher than that of Teth39 AKR with isobutyraldehyde and 44.2-fold higher with butyraldehyde. Expression of Teth39 *bdhA* might therefore alternatively be used to biologically produce these higher energy fuel compounds in thermophilic microbes.

## Conclusions

Inhibitors, such as furfural and 5-HMF, are generated from common pretreatment methods used for improving the reactivity of lignocellulosic biomass toward enzymatic solubilization. The physiological response and tolerance to these inhibitors must be understood in order to develop improved microorganisms such as *C. thermocellum* or a *Caldicellulosiruptor* sp. for thermophilic consolidated bioprocessing of biomass. We measured rapid growth in the presence of 10 to 15 mM furan aldehydes and their detoxification *in situ* by a thermophilic anaerobe, *T. pseudethanolicus* 39E. Physiological effects resulting from higher furfural and 5-HMF concentrations included decreased ethanol yield with increases in acetate and lactate production from glucose. A bottom-up proteomics approach was applied to screen for potential enzymes or pathways directly involved in furan aldehyde detoxification. While a number of cellular functions were impacted, including a decrease in expression of ADHs involved in ethanol production, an ADH encoded by Teth39_1597 was upregulated nearly sevenfold in the presence of 15 mM furfural. The enzyme, a putative butanol dehydrogenase, was cloned and overexpressed in *E. coli* and displayed NADPH-dependent activity against furfural and 5-HMF, suggesting a direct role in detoxifying furan aldehyde inhibitors *in situ*.

## Methods

### Growth experiments

*Thermoanaerobacter pseudethanolicus* 39E (DSMZ 2355) was purchased from Deutsche Sammlung von Mikroorganismen und Zellkulturen GmbH (DSMZ, Braunschweig, Germany). All chemicals were purchased from Sigma-Aldrich (St. Louis, MO) unless otherwise indicated. The anaerobic growth medium was prepared using a modified Hungate technique [48] and consisted of 4.5 mM KCl, 4.7 mM NH_4_Cl, 2.5 mM MgSO_4_ ∙7H_2_O, 1.0 mM NaCl, 0.7 mM CaCl_2_ ∙2H_2_O, 0.25 mg/mL resazurin, 2.8 mM cysteine-HCl, 6.0 mM NaHCO_3_, 1 mM potassium phosphate buffer (pH 6.8), 10 mM 3-(N-morpholino)propanesulfonic acid (pH 6.8), 1x Wolfe’s trace minerals [49], 1× Wolfe’s vitamin supplement [49], 0.1% (w/v) yeast extract (Fisher Scientific, Pittsburgh, PA), and 40 mM glucose. Furan aldehydes were added from degassed concentrated stock solutions. Cultures were grown at 65°C from a 1% inoculum in Balch tubes (10 mL) or 125-mL serum bottles (50 mL). Cell growth was monitored by optical density at 600 nm, either directly in the Balch tube using a Spectronic 200 spectrophotometer (Thermo Fisher Scientific, Waltham, MA) or as 200-μL samples transferred to a 96-well plate and read on a Synergy Mx plate reader (BioTek, Winooski, VT). All growth experiments were performed in triplicate.

### Small molecule measurement

Glucose, lactate, acetate, and ethanol were measured via HPLC as previously described using an HPX-87H column (Bio-Rad Laboratories, Hercules, CA) at 60°C with detection via refractive index and 5 mM H_2_SO_4_ as the mobile phase [50]. Furfural and 5-HMF were measured spectrophotometrically (DU 800, Beckman Coulter, Brea, CA) at 304 and 323 nm, respectively, and concentrations were determined using standard curves generated in growth medium. 2,5-furandimethanol was measured using gas chromatography-mass spectrometry (GC-MS) following trimethylsilylation, with an Agilent 5975C standard quadrupole GC-MS using electron impact ionization (970 eV), as described previously [51].

### Proteomic analysis: sample preparation

Cell pellets (10 to 50 mg) from cultures grown for 8 h (early stationary phase) with and without 15 mM furfural were frozen at -80°C prior to preparation. The thawed pellets were resuspended in 1 mL lysis buffer (4% SDS, 100 mM Tris-HCl pH 8.0, 50 mM dithiothreitol) and boiled for 5 min. Samples were then pulse-sonicated (10 s on, 10 s off) for 2 min with an ultrasonic disruptor (Branson, Danbury, CT) at 20% amplitude. The samples were boiled again for 5 min, cleared by centrifugation (21,000 × *g*, 10 min, RT), and immediately precipitated with 20% trichloroacetic acid overnight at -20°C. Precipitated proteins were washed twice with ice-cold acetone, air dried, and resuspended in 8 M urea in 100 mM Tris-HCl, pH 8.0. The samples were sonicated as before and incubated for 30 min at room temperature (RT). Samples were adjusted to 10 mM dithiothreitol (10 min, RT) and then 10 mM iodoacetamide (10 min, RT, in the dark) to both reduce and block cysteine residues. Sample aliquots containing about 1.5 mg of crude protein were diluted 1:1 (v/v) with 100 mM Tris-HCl, pH 8.0 and 20 mM CaCl_2_ and digested with sequencing-grade trypsin (Promega, San Luis Obispo, CA) at a 1:75 (w/w) enzyme:protein ratio (16 h, RT). The samples were again diluted 1:1 (v/v) and digested with a second aliquot of trypsin (1:75; w/w) for an additional 4 h. Following digestion, each sample was adjusted to 200 mM NaCl and 0.1% formic acid and filtered through a 10-kDa cutoff spin column filter (Vivaspin 2, GE Healthcare, Pittsburgh, PA). The peptide-enriched flow-through was then quantified by the bicinchoninic acid assay.

### Proteomic analysis: measurement of peptides by two-dimensional liquid chromatography-tandem mass spectrometry (LC-MS/MS)

For each sample, 100 μg of peptides were bomb-loaded onto a biphasic MudPIT back column [52] packed with about 5 cm of strong cation exchange (SCX) resin for charge-based separation of peptides followed by about 3 cm C18 reversed phase (RP) for online desalting (Luna and Aqua respectively, Phenomenex, Torrance, CA). Once loaded, the sample columns were washed offline with solvent A (5% acetonitrile, 95% HPLC-grade water, 0.1% formic acid) for 15 min, followed by a gradient to 100% solvent B (70% acetonitrile, 30% HPLC-grade water, 0.1% formic acid) over 30 min. The washed samples were then placed in-line with an in-house pulled nanospray emitter (100-μm inner diameter) packed with 15 cm of C18 RP material and analyzed via 24-h MudPIT two-dimensional LC-MS/MS (eleven salt pulses: 5, 7.5, 10, 12.5, 15, 17.5, 20, 25, 35, 50, 100% of 500 mM ammonium acetate followed by a 100-min gradient to 50% solvent B) with an LTQ XL mass spectrometer (Thermo Fisher Scientific) operating in data-dependent mode. A total of three biological replicate measurements were obtained for each sample.

### Proteomic analysis: MS data analysis and evaluation

Acquired MS/MS spectra were assigned to specific peptide sequences using the SEQUEST search algorithm [53] with a FASTA proteome database specific to *T. pseudethanolicus*. The database contained common contaminant protein entries as well as reversed decoy sequences to assess protein-level false discovery rates. SEQUEST-scored peptide sequence data were filtered and assembled into protein loci using DTASelect [54] with the following conservative criteria: XCorr: +1 = 1.8, +2 = 2.5, +3 = 3.5, DeltCN 0.08, and two peptides per protein identification with at least one required to be unique.

Prior to the semiquantitative analysis, spectral counts were rebalanced to properly distribute non-unique/shared peptides between their potential parent proteins, as previously described [55]. To represent proteins that were sporadically identified across runs (that is, blank/zero values in a portion of the six sample runs), a fraction of a spectral count (0.33) was added to the entire dataset. This distributional shift maintains the originally measured spectral count differential but allows for blank/zero values to be considered in the ensuing statistical analysis [56]. These adjusted values were then converted to normalized spectral counts (nSpC), an extension of the widely recognized normalized spectral abundance factor (NSAF) [57] that is calculated by multiplying the NSAF values by an arbitrary number representative of the number of spectra collected for each run. In this case, the number 50,000 was used for facile data interpretation. Once calculated, an SpC cutoff was applied to all proteins identified in the dataset so that 99% of the total raw SpC assigned to each (summed across all replicates and conditions) remained.

These remaining proteins were log2 transformed, and statistically assessed by ANOVA with JMP Genomics ver. 4.1 (SAS Institute, Cary, NC) to identify proteins in the furfural treated samples that were significantly (*P* ≤0.05) up- or downregulated relative to the control. These differentially expressed proteins were then hierarchically clustered based on their abundance patterns across all replicates and conditions using the “Fast Ward” algorithm. To remove differences based on raw magnitude differences in nSpC, each protein’s abundance was standardized to represent the number of standard deviations away from the row mean.

### Aldehyde reductase cloning and overexpression

*T. pseudethanolicus* genomic DNA was isolated using the Wizard Genomic DNA Purification kit (Promega, Madison, WI). Teth39_1597 and Teth39_1598 were PCR amplified using Phusion Polymerase (New England Biolabs, Ipswich, MA), cloned into pET-30a (EMD Millipore, Billerica, MA) behind 6xHis- and S-tags, and the final constructs were sequence verified (University of Tennessee, Knoxville, Molecular Biology Resource Facility). Expression plasmids were transformed in BL21 (DE3) *Escherichia coli* according to the manufacturer’s protocol (Invitrogen, Grand Island, NY). Cells were grown in 50 mL 2×YT medium at 37°C to OD_600_ of 0.8-1.0, then induced with 100 μM isopropyl-β-D-thiogalactopyranoside (IPTG) and switched to 30°C for 16 h. Cells were harvested at 4°C (3,000 × *g*, 30 min), washed in 50 mL 100 mM sodium phosphate buffer, pH 7 (buffer A), and resuspended in 5 mL buffer A. The cell suspension (450 μL) was added to 0.1 mm zirconia beads (300 μL) and vortexed 4 × 60 s with 30 s on ice in between. The samples were centrifuged (14,000 × *g*, 2 min), and the resulting supernatant was used for enzyme assays.

### Enyzme assays

The *in vitro* aldehyde reductase activity was measured as previously described [23] in 100 mM sodium phosphate buffer (pH 7) with 0.2 mM NAD(P)H and 20 mM substrate. The assay mix was added to a sealed 2-mL quartz cuvette and equilibrated to 60°C. Assays were read at 340 nm (DU 800) for 150 s to establish a baseline slope before whole cell lysate was added (1 to 5 μL). The cuvettes were inverted once to mix and read an additional 450 s. The decrease in absorbance over time was calculated and the baseline slope was subtracted. The NAD(P)H concentration was determined using the extinction coefficient (NADH: 6,220 M^-1^ cm^-1^, NADPH: 6,270 M^-1^ cm^-1^), and the specific activity was calculated as the change in μmol NAD(P)H/min/mg of whole cell lysate protein. Protein concentration was determined using the Bradford assay (Bio-Rad, Hercules, CA) with bovine serum albumin as a standard. The specific activity was measured for the pET-30a vector (control) and overexpressed Teth39_1597 and Teth39_1598 with acetaldehyde, butyraldehyde, isobutyraldehyde, furfural, and 5-HMF.

## Additional file

Additional file 1: Table S1. Description of data. Proteomics data file for 225 proteins that were differentially regulated in response to a 15 mM furfural treatment. Color-coded hierarchical cluster analysis, cluster number, cluster order, average nSpC per condition, fold change, and *P*-value are provided for all replicates. The raw output for all detected proteins and statistical analyses are also included.

## Abbreviations

5-HMF: 5-hydroxymethylfurfural
ADH: alcohol dehydrogenase
AKR: Aldo/keto reductase
ANOVA: analysis of variance
BDH: butanol dehydrogenase
CBP: consolidated bioprocessing
GC-MS: gas chromatography-mass spectrometry
HPLC: high performance liquid chromatography
IPTG: isopropyl-β-D-thiogalactopyranoside
LC-MS: liquid chromatography-mass spectrometry
nSpC: normalized spectral counts
OD: optical density
RP: reverse phase
SpC: spectral counts
TCA: tricarboxylic acid
